# Glowing gels and pipettes aplenty: how do commercial stock image banks portray genetic tests?

**DOI:** 10.1038/s41431-023-01508-4

**Published:** 2023-12-08

**Authors:** Rachel Horton, Leah Boyle, Susie Weller, Anneke Lucassen

**Affiliations:** 1 Clinical Ethics, Law and Society Group, University of Oxford, Oxford, UK; 2 Centre for Personalised Medicine, St Anne’s College, University of Oxford, Oxford, UK; 3 Wessex Clinical Genetics Service, Princess Anne Hospital, Southampton, UK; 4 Cancer Epidemiology Unit, Oxford Population Health, University of Oxford, Oxford, UK

**Keywords:** Ethics, Medical ethics

## Abstract

News stories and patient-facing material about genetic tests are often illustrated by images, but the content of such images and the messages they propagate are rarely scrutinised. Stock image banks were searched to identify a hundred images relating to genetic tests and analysed using a multimodal critical discourse approach, aiming to identify what the images featured, how they were composed, and what they communicated about genetic testing. We found that images tended to focus on technical aspects of sample processing (for example, pipetting) and drew on older technologies (for example slab gel electrophoresis) when representing data arising from genetic tests. Composition choices like focussing images around pipette tips, or emphasising colour or brightness of electrophoretic bands, represented genetic testing as precise, unambiguous and illuminating. Only 7% of images featured a person having a genetic test, and only one image alluded to communication of genetic results. Current popular visual representations of genetic testing rarely highlight the possibility of uncertain or non-diagnostic outcomes, and may contribute to high public expectations of informativeness and certainty from such tests.

## Introduction

It is often said that a picture is worth a thousand words, but analysis of popular discourse around genetic testing has, in recent years, tended to focus on text [1, 2]. However, newspapers, online articles, social media stories, and patient leaflets about genetic testing are often illustrated by images. For people who do not go on to read the associated text, a headline and its associated image may be all they see when browsing news applications or scrolling through social media, and hence, the images featured may have an important role in shaping their expectations of and ideas around genetic tests. Stories about genetics in healthcare sometimes feature specific people or events (for example, the coverage of Angelina Jolie’s *BRCA* testing [3], or people’s experiences with direct-to-consumer genetic tests [4]), but many tend to focus more broadly on genetic testing initiatives or outcomes arising from these. As such they may lend themselves to the use of stock images [5].

In this article, we analyse the messages that commercial stock images convey about genetic tests. Commercial stock image banks buy images that they then sell on for use by clients in an industry estimated to be worth 3.3 billion US dollars in 2020 [6]. The more times a stock image is used, the more lucrative it will be, meaning that successful stock images need to be both applicable to many contexts, and feel tailored to the particular context in which a client might be looking to use them. As Frosh writes, ‘to borrow an analogy from the garment trade, a successful image needs to look off-the-shelf and tailor-made’ [7]. Machin discusses how the most lucrative stock images conform with existing and somewhat problematic clichés: ‘will we still be able to recognise ‘work’ without the laptop, ‘freedom’ without someone jumping, and ‘ethnicity’ without bright and multi-coloured clothing?’ [8].

The imagery around genetics was subject to significant attention in the 1990s and 2000s, focussing on the mental pictures being constructed as genetic science evolved. In Imagenation, written in 1998, Van Dijck reflects on the reification of genes and societal tendency to view them as separate from the context in which they operate. She notes a number of instances where visual images serve to propagate such ideas: ‘The double helix – once a model for the structure of DNA – surfaces in mass media as a self-explanatory icon of genetic determinism. Covers of magazines feature scientists wrapped in double helical strings as politicians wrap themselves in the flag. The two spiralling base-chains have come to be commonly associated with medical progress, signalling universal hope for curing congenital and other disease’. Regarding the depiction of people involved in the genetics world, Van Dijck describes how molecular biologists and geneticists are typically ‘dressed in white lab coats and surrounded by sophisticated high-tech equipment… often paired off with colourful enlargements of microscopic images that make infinitesimal molecules visible to the ordinary eye’. She reflects on parallels commonly drawn between space exploration and genetics: ‘geneticists are repeatedly referred to as ‘astronauts’ of the new science, and genetics as a space adventure’ [9].

The DNA Mystique, written by Nelkin and Lindee in 1997, similarly considers how ‘The double helix has… become a playful icon in architecture, education, and commercial culture. DNA, invisible in real life, often appears as a supersized helical structure, as if to testify to its larger-than-life properties’. The authors reflect on how such uses tend to fuel and reinforce genetic essentialist views and discuss how ‘DNA has assumed a cultural meaning similar to that of the Biblical soul’ [10].

Recently, analysis of imagery around genetics has tended to examine representation of controversial technologies with a genetic basis, such as cloning or germline editing [11], or the development of ‘sciart’ relating to genetics and genomics. In The Genome Incorporated, written in 2010, O’Riordan discusses the rise of ‘genomic portraits’ (the incorporation of a person’s genetic material into art), which can be seen to equate ‘DNA with the notion of the truth of the person’, and recognises gel electrophoresis-based pictures as a common feature of imagery relating to genetics: ‘DNA bands or ladders across a surface resembling something like a photographic negative has become a distinctive and frequently used genomic icon’ [12].

Since such works were published, clinical genetic testing has evolved substantially, and contemporary genetic tests vary greatly in the solidity and significance of their outcomes. Some tests allow (relatively) clear predictions about a person’s health—for example, predictive testing for Huntington’s disease. For some tests, while at the variant level the implications may be clear, the likely consequences for the person over their lifetime may be heavily context-dependent, for example being heterozygous for a ΔF508 variant in *CFTR* [13]. For other tests, predicting a person’s health from genetic data may be very challenging, for example determining the significance of a rare non-synonymous variant in a developmental disease gene in a seemingly healthy newborn [14].

As clinical practice increasingly shifts from targeted genetic testing to broad genomic approaches, we wanted to explore the ways in which stock images portray genetic tests, and how the range of possible outcomes available from testing is reflected in such images. We found that criticisms made of genetic imagery in the 1990s are still highly relevant today. Though technology used in genetic testing, and access to testing, has evolved considerably, the ways in which ‘genetic testing’ is represented in stock image banks seems stuck in the past, with a predominant emphasis on the technical nature of sample processing and an illuminating readout.

## Methods

### Research design

We used a multimodal critical discourse approach, an analytic stance that focusses on communicative practice [15, 16]. We were particularly influenced by Harvey and Brookes, the first to examine critically the visual discourses around a health topic (dementia) in a stock image bank. Here, stock images are considered as ‘visual texts culminating from a system of deliberate and motivated design choices made by their producers’, and analysed via a two-tier approach, focussing first on the content and presentation of the images, then on the messages that these stylistic choices convey [5].

### Data generation

We identified images using Getty images and Adobe Stock, image banks chosen on the basis of their size, popularity and longevity, using the search term ‘genetic test’ on 8/11/22. Within each image bank, we ranked search results according to relevance/best match (inbuilt search filters provided by Adobe Stock and Getty images, respectively). We previously piloted ‘most popular/most downloads’ and found that many images were generic laboratory scenes, likely frequently used because these images were potentially applicable to many more stories, and so their popularity could not be taken as meaning they were more likely to be chosen to illustrate a story about genetic testing. We selected the 50 most relevant or ‘best match’ images from each image bank for analysis, as high ranking images would be most likely to be seen by people seeking images to illustrate their work (for example, for Google searches, most people follow links on the first page of results only [17]).

### Image analysis

Having identified the stock images, two researchers (RH and LB) looked separately at each image and documented their features firstly using free text, for example: what is depicted? What is the setting? Are there people in the image and if so, who are they, how much of them is visible, where is their gaze directed? How is the image lit and what colours are used? Secondly, RH and LB identified objects frequently featured in the images (pipettes, microscopes, the double helix, bands from slab gel electrophoresis) then went systematically through every image specifically noting whether such objects were present or absent. We then reviewed the images within each category (e.g., all images featuring a pipette or dropper) aiming to identify common features as to how these were presented (e.g., was there a droplet at the end of the pipette or dropper? Was the tip of the pipette or dropper at the centre of the image?), before counting what proportion of images this applied to. At various points throughout the course of this research, we discussed our developing findings within our authorship group and wider research group (Clinical Ethics, Law and Society, University of Oxford). During these discussions, we considered what the features we identified might express about genetic testing, looking at how genetic tests are symbolised visually, and considering the messages that these might create or propagate regarding genetic testing [5].

## Results

Of the 100 images reviewed, most were strikingly clean and crisp, likely increasing their attractiveness for use as illustrations, but potentially also sculpting an expectation of neatness and clarity from genetic tests. See Table 1 for a summary of the images identified.

**Table 1:** Overview of images identified via stock image bank search.

Primary process shown	Number of images	Examples
Taking a sample for genetic testing	9	Person having cheek swab Saliva collection tube from commercial testing kit Sample of hair held up by tweezers Person in scrubs holding swab Person in lab coat holding up filled blood collection tube
Processing a sample for genetic testing	32	Pipetting Rack of test tubes or Eppendorfs
Data from genetic testing/ analysing data from genetic testing	14	Image based on slab gel electrophoresis Person in lab coat looking at computer screen, image from slab gel, or looking down microscope Microscope lens pointing at a slide
Processing a sample AND data from genetic testing	20	Pipette held over petri dish lying on picture from slab gel electrophoresis Eppendorf or sample tube held up in front of picture from slab gel electrophoresis Person in lab coat pipetting next to microscope and/or monitor showing double helix and bar charts
Delivery of results from genetic testing	2	Couple sat holding hands (faces not in picture), hands of person in white coat holding clipboard visible Positive COVID lateral flow test
Other	23	Double helix (sometimes with e.g., magnifying glass held in front of it, people in white coats peering at it, figures around it holding bottle of pills etc) Dot-and-line diagrams implying molecular structures or constellations

### Bench work in genetic testing

‘Wet lab’ aspects of genetic testing were prominent in the image banks, with many images illustrating different stages of sample processing. Images included various scientific equipment emphasising technical aspects of genetic testing. For example, 19% of images included a microscope, potentially evoking ideas of scrutiny and detailed examination, despite microscopes not being prominently involved in most genetic tests. This echoed Van Dijck’s observation in *Imagenation* regarding the ‘ostentatious presence of… visualising instruments’ in pictures of genetic scientists in the 1990s [9].

**Fig. 1:**
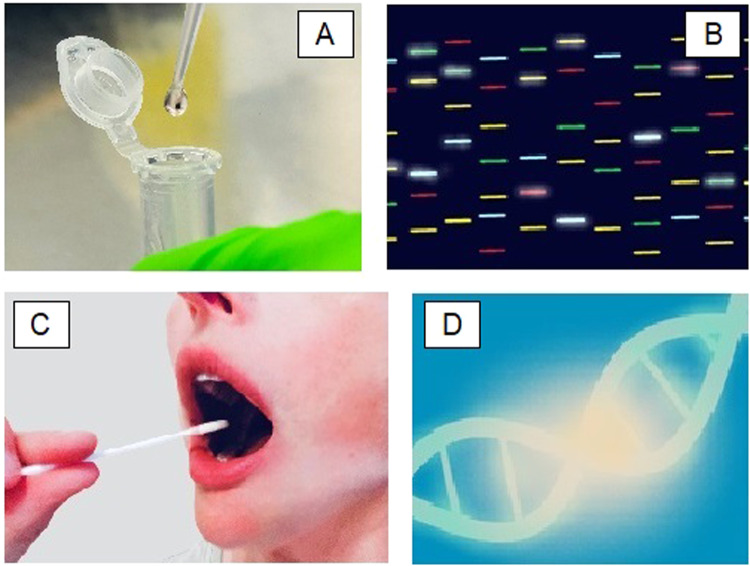
Features of stock images relating to genetic tests. **A** 39% of images featured a pipette or dropper. **B** Slab gel electrophoresis pictures were often highly coloured with glowing bands. **C** Only 7% of images featured someone having a genetic test. **D** 30% of pictures featuring a double helix had light shining out of it. For copyright reasons, this figure uses pictures intended to be representative of the images analysed, rather than including original images from the image bank search.

Samples being tested were represented with varying degrees of relatedness to actual practice, ranging from barcoded vacutainer blood collection tubes and Eppendorfs containing small volumes of colourless fluid at one end of the spectrum, to large conical and round-bottom flasks filled with bright blue and orange liquids at the other. Pipettes and droppers featured in 39% of the images, as represented in Fig. 1A, and were often prominent, for example in 18% of the images analysed (46% of the images featuring a pipette), the focal point was a droplet of liquid suspended at the end of a pipette or dropper, potentially evoking ideas of precision, accuracy and immediacy as the droplet stands ready to fall.

### Analysis of genetic data

24% of stock images included a slab gel electrophoresis picture of some sort, supporting O’Riordan’s 2010 observation that DNA ‘bands’ or ‘ladders’ constitute a ‘genomic icon’ [12]. There was a marked discrepancy between the image banks here in that images involving gels comprised 42% of the Getty images but only 6% of Adobe Stock (though a further 6% of the Adobe Stock images included ‘band-like’ motifs such as might be seen on a gel). Across both image banks, 60% of images involving gels showed multiple different coloured bands, and in 46% of images featuring gels, bands were noticeably glowing, as represented in Fig. 1B. This depiction of brightly coloured objects against a dark background was reminiscent of parallels that popular texts sometimes draw between genetics and space exploration [9].

Most gel pictures looked like they had originally been based on a restriction fragment length polymorphism assay or similar. The choice to lean on this older technology in creating stock images, showing precise (often glowing) barcode-like bands, arguably tended to represent genetic tests as clear, unambiguous and illuminating. None of the images representing data for analysis alluded to widely-used more recent technologies, for example, there were no images featuring nucleotides as letters ACGT, and no images featuring e.g., read alignment viewers or data from VCF files. This likely partly reflects our choice to search the image banks for ‘genetic test’ as opposed to ‘genomic test’ (as genetic is a more widely understood term [18]). However, an informal search of both image banks for the term ‘genomic test’ indicates that gel-based pictures still predominate relative to, for example, ACGT-based representations of data.

### Absent patients and shadowy scientists

Only 7% of images included people who themselves were having genetic tests. Where enough of the person was shown in the photograph to make an assessment, they all appeared to be young adults. Many images showed very little of the person or portrayed them in a passive and vulnerable position, for example, three images showed a woman having a cheek swab (represented in Fig. 1C). Only one image alluded to communication of results from a genetic test: a woman and man were pictured in casual clothes sat together holding hands, while visible to their left were the arms of a third person wearing a white coat, writing on a clipboard on which rested a stethoscope. The faces of the couple were not included in the picture, echoing the tendency reflected in the stock images overall to edit the people undergoing genetic testing, out of the images that represent genetic tests.

In contrast, people involved in delivering genetic tests were pictured in 59% of images, typically wearing clothes that emphasised a scientific/technical role, for example, white coats, blue gloves, safety glasses or goggles. However, in most images the predominant focus was not on the person themselves—for example, in 41% of images featuring people working with genetic tests, only hands or fingertips were visible, and while faces were featured in 42% of the images featuring people working with genetic tests, there were no instances where a person was looking directly at the camera. While people were illustrated as being involved in processing genetic tests, primacy seemed typically to be given to the equipment or samples they were holding or engaging with. The role that people might have in delivering results, or exploring what these might mean in the context of an individual’s life, was rarely acknowledged.

### The double helix

The double helix featured in 23% of the stock images analysed. Again, there was an imbalance between the different image banks, with 42% of Adobe Stock images featuring the double helix versus only 4% of Getty images. Where an image included a double helix, this tended to be prominent or the focal point of the image: in 30% of images featuring a double helix, this was emphasised by glowing or sparkling light seeming to emanate from the helix, as illustrated in Fig. 1D. The use of the double helix to illustrate genetic test stock images taps into connotations around the discovery of its structure, which was heralded as ‘the secret of life’ [19, 20]. Its use in images illustrating genetic testing may serve to emphasise that such tests directly examine something fundamental, and potentially invites an expectation that insights will shine directly out of the analysis in the same way that light emanates from the helix in these pictures. As Nelkin and Lindee discuss in ‘The DNA Mystique’, such depictions may serve to ‘glamorize DNA and promote the notion of genetic essentialism’ [10].

## Discussion

In many different settings, ranging from mainstream media to policy documents, texts discussing genetics are illustrated with images. These images may influence people’s perceptions of genetic testing. This is obviously not an exhaustive analysis of how genetics is visually represented in the media, and images were examined separate to the context of their use. We have not studied public perceptions as to the ideas evoked by these images and clearly these may differ from our own interpretations, and will be an important question to consider in future work. Nevertheless, it is striking that stock image banks tend to rely heavily on symbols that reinforce the idea of genetic testing as precise, clear and illuminating, and that these symbols do not appear to have been updated to keep pace with shifting genetic technology.

Images emphasise technical aspects of sample preparation, and continue to draw on products of older technologies, such as slab gel electrophoresis, to present genetic analysis as akin to reading a barcode. The uncertainties and challenges that genetic tests can sometimes create are rarely made visible, perhaps in part because this messiness and complexity are hard to capture in a single image. The role of people in having, creating, and living with genetic tests is obscured – patients are rarely pictured and scientists and clinicians play second fiddle to the technical paraphernalia involved in sample processing. Questions raised in the 1990s as to the helpfulness of common imagery around genetics are still highly relevant today, with many images in common use potentially feeding into expectations that testing will deliver a clear cut, illuminating and powerful readout from our genetic code.

The images used to illustrate news stories, leaflets, press releases and journal covers about genetics are not just pretty pictures, they communicate messages about genetics whether we intend them to or not. Some of the images we analysed may invite people to expect more clarity and brighter insights from genetic testing than will often be the case: many people with rare diseases remain without a genetic diagnosis [21], and many genetic tests generate uncertainty, or understanding of their meaning evolves over time [22]. In public speaking training we often hear that only a small fraction of what people take away from our talks is the content – how we sound and look also tell a story. Similarly, the pictures we choose to illustrate text about genetic testing may seem like an afterthought but it is worth reflecting on what they actually communicate.

Van Dijck challenges us that ‘Genome researchers are not at the mercy of journalists for crafting their public images, but play an essential role in the crafting process… the ‘image’ of genetics is produced simultaneously by scientists, journalists and public relations managers. They continuously occupy each other’s terrain, and all draw on the same discourse of information’ [9]. Condit echoed an important role for geneticists in influencing popular discourse in her 2007 article ‘How geneticists can help reporters to get their story right’, reflecting on the quandary science reporters may experience between attracting interest and attention for their article and presenting information dispassionately and ‘objectively’, and the challenges of presenting complex and nuanced stories in an accessible format. While the pointers Condit gave in the article relate to conversations with journalists (reducing hype; avoiding determinism; countering discrimination) [23], these are also relevant to the selection of images to illustrate work relating to genetics.

The 2006 Wellcome-funded publication Engaging Science: Thoughts, deeds, analysis and action, reflected on the role of public engagement, making the point that this should not serve purely ‘to explain and promote science’, but to facilitate ‘a discriminating populace able to exercise their own judgement on topics from stem cells to nuclear energy’. Within this report, Scheufele makes the point that in developing opinions around scientific advances we tend to be ‘cognitive misers’ and rather than gleaning extensive information from multiple sources in order to come to an opinion, people instead tend to seek only as much as they feel they need to make a given decision. The framing of the issues under discussion is, therefore, very important, and visual images may make a significant contribution to this. Indeed, later in the same report, Kitzinger discusses research that ‘highlights the importance of visuals or narrative structure over the surface logic of any particular media text’ in what people take away from TV programmes or news reports [24].

Finding alternative ways to visually represent genetic testing is challenging, and we frequently turn to the sparkly blue double helix as a shortcut to illustrate texts, presentations and podcasts—it looks good and gives the viewer a clear cue that the content will relate to genetics. But are there other representations we might usefully bring into conversations about genetic tests? We aim to explore this in future research, working with people having and providing such tests.

## Data Availability

The images analysed in this article are available upon request.
